# A case of acute necrotizing pancreatitis complicated with non ST elevation myocardial infarction

**DOI:** 10.1186/s13104-018-3274-0

**Published:** 2018-03-09

**Authors:** Udaya Ralapanawa, Thilak Jayalath, Dhanusha Senadhira

**Affiliations:** 1 Faculty of Medicine, University of Peradeniya, Peradeniya, Sri Lanka; 2 Teaching Hospital Peradeniya, Peradeniya, Sri Lanka

**Keywords:** Acute pancreatitis, Myocardial infarction, 2D echocardiogram, ECG, Troponin I

## Abstract

**Background:**

Acute pancreatitis is an inflammatory condition with varying severity and a range of local and systemic complications. Here we report a patient with acute necrotizing pancreatitis complicated with a true non ST elevation myocardial infarction.

**Case presentation:**

A 58 year old lady was admitted to our unit with acute onset epigastric pain and vomiting for 4 h duration. Following admission she complained of retrosternal tightening type of a chest pain. She had elevated serum amylase and cardiac troponin. Electrocardiogram (ECG) revealed lateral ischaemia. Contrast computerized tomography abdomen revealed acute severe necrotizing pancreatitis.

**Conclusions:**

Nonspecific ECG changes can occur in patients with acute pancreatitis. But the diagnosis of true myocardial infarction in a context of acute pancreatitis using ECGs, 2D echocardiography, cardiac biomarkers and coronary angiograms can be challenging with the choice of revascularization therapy and safety of antiplatelet agents and anticoagulant therapy. Decision making regarding the management of such a patient is also critical.

## Background

Acute pancreatitis is an inflammatory condition of pancreas which can range in severity from mild to severe disease. Most patients develop self-limiting disease but a minority of patients progress to a severe form with both systemic and local complications. Cardiovascular complications of acute pancreatitis include shock with systemic inflammatory response syndrome, hypovolemia, pericardial effusions and non-specific ST segment changes. Here we report a case of 58 year old lady who developed a non ST elevation myocardial infarction as a complication of acute necrotizing pancreatitis.

## Case presentation

**Fig. 1:**
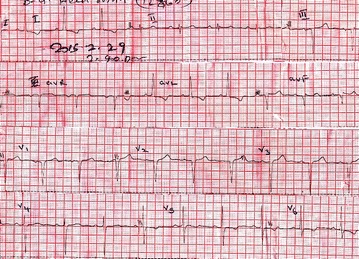
12 lead ECG on admission

**Fig. 2:**
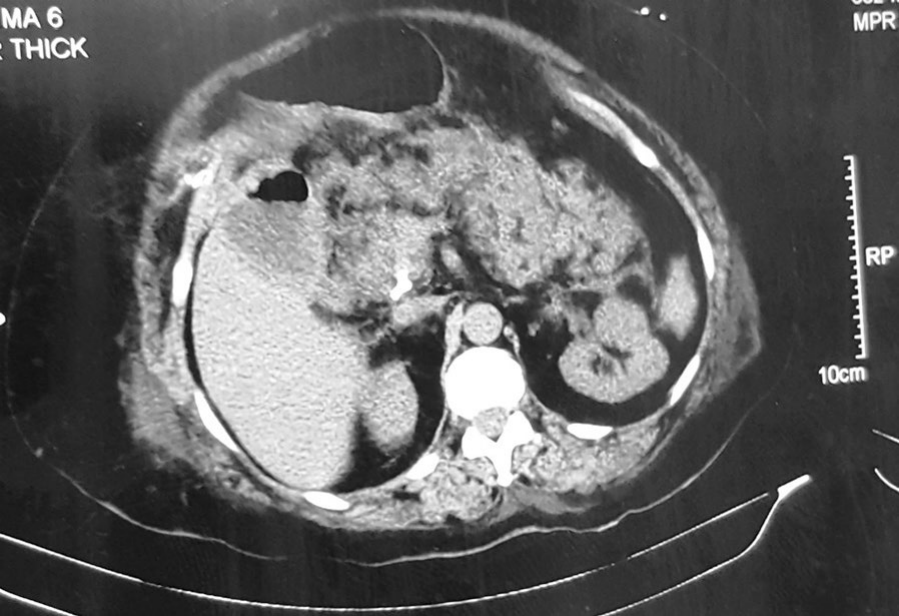
CT abdomen showing necrotizing panceatitis

A 58 year old lady was admitted to our unit with acute onset epigastric pain and vomiting for 4 h duration. She is a diagnosed patient with type 2 diabetes mellitus for 10 years and hypertension for 5 years. Her diabetes was well controlled with HbA1c of 6 and without significant micro or macrovascular complications. She is a nonalcoholic. Following admission she complained of retrosternal tightening type of a chest pain suggestive of acute coronary syndrome. Pain was sudden onset, lasted for 20 min in moderate intensity and responded to the medications given in hospital. Physical examination revealed severe epigastric tenderness. There was no rebound tenderness. Cardiovascular and respiratory examinations were normal. She was haemodynamically stable with a blood pressure of 130/90 mmHg and pulse rate of 96 bpm. 12 lead electrocardiogram (ECG) revealed T wave inversions in lateral leads including I, aVL, V5 and V6 (Fig. 1). Her serum troponin I titer was 14 ng/mL (normal less than 0.8 ng/mL). 2D echo revealed lateral wall hypokinesia with preserved left ventricular function. Her serum amylase level on admission was 1741 U/L (20–115 U/L). Contrast computerized tomography (CT) abdomen revealed acute severe necrotizing pancreatitis without pseudocyst formation (Fig. 2). Serum calcium, creatinine phosphokinase (CPK) and triglyceride levels were normal. Therefore she belongs to the moderately severe acute pancreatitis according to revised Atalanta criteria (2013). She was free of chest pain and haemodynamically stable following starting treatment with aspirin, clopidogrel, atorvastatin and nitroglycerin. Therefore she was given unfractionated heparin for 72 h and a coronary angiogram planned to be done later. Her serial ECGs showed minor dynamic T wave changes. She was given intensive care and improved during next few days with standard management protocol of acute pancreatitis. Three days later her repeat serum troponin level remained at 9.2 ng/mL and amylase level was 504 U/L. A coronary angiogram was done after 2 weeks. It revealed minor coronary artery disease with only 20–30% stenosis at proximal left anterior descending artery.

## Discussion and conclusion

Electrocardiographic changes are relatively common in acute pancreatitis. They include tachy and brady arrhythmias, conduction abnormalities including bundle branch blocks and changes in T wave and QT period [1]. These abnormalities are seen in approximately 50% of patients [2]. Experimental studies have reported that in acute pancreatitis there are myocardial ultra-structural disturbances including interstitial edema and cardiomyocyte hypoxia, myofibril over activity, intercellular oedema between cardiomyocytes and cardiomyocyte hypertrophy with collagenation of myocardial stroma [3, 4]. Elevated levels of cardiac troponins can be seen among patients with acute pancreatitis without true myocardial infarctions [5]. This can be attributable to rhabdomyolysis which is associated with acute pancreatitis [6]. In a study on the determination of myoglobin in acute pancreatitis patients has shown that 20% of acute pancreatitis patients had serum myoglobin concentrations above the upper normal limit [7].

Acute pancreatitis associated with true myocardial infarction is a rare incident and Kumara et al. [8] have reported such a true ST elevation myocardial infarction in a young patient with acute pancreatitis. In 2005, Krantzopoulos et al. [9] also have reported another case of myocardial infarction with acute pancreatitis. Diagnosis of acute myocardial infarction among patients with acute pancreatitis can be difficult. Erroneously administered thrombolytic therapy for pseudo myocardial infarction patients can have disastrous outcome. Cafri et al. [10] reported a 54 year old male who underwent thrombolytic therapy after being misdiagnosed with myocardial infarction. Precise mechanism of myocardial infarction in acute pancreatitis remains unclear. Hypothesized mechanisms for ECG changes and myocardial infarction include vagally mediated reflexes [11], metabolic and electrolyte abnormalities [12] toxic effect of pancreatic enzymes on myocardium, coronary artery spasm and prothrombotic derangement.

Above described patient’s main presentation was acute pancreatitis. Also she is a known patient with type 2 diabetes mellitus and hypertension. In that context she has developed a true myocardial infarction as evidenced by high troponin I level and echocardiographic evidence of lateral wall hypokinesia. Although high troponin levels can occur with rhabdomyolysis among acute pancreatitis patients, this patient’s CPK level was normal. We had no facilities to carry out an urgent coronary angiogram at out Centre. Considering her haemodynamic stability and absence of ongoing chest pain we did coronary angiogram later and it revealed only 20–30% stenosis at proximal left anterior descending artery. Although she had risk factors for coronary artery disease, this episode of non ST elevation myocardial infarction cannot be attributable to atherosclerotic disease. That is because of her angiogram findings. Therefore most probably her non ST elevation myocardial infarction can be considered to be related with acute pancreatitis (Table 1).

**Table 1:** Summary of the recent literature review on cases with acute pancreatitis complicated with acute coronary syndrome (Source PubMed)

Title	Authors	Date of publication
Case series of pancreatitis with uncommon presentations	Redkar NN, Rawat KJ, Tiwari D, Panandikar G	2015
Acute pancreatitis complicated by acute myocardial infarction—a rare association	Vasantha Kumar A, Mohan Reddy G, Anirudh Kumar A	2013
Acute pancreatitis complicated by ST-elevation myocardial infarction	Phadke MS, Punjabi P, Sharma S, Kide S, Nawale J, Chaurasia A	2013
Myocardial infarction as complication of acute pancreatitis	Asfalou I, Miftah F, Kendoussi M, Raissouni M, Benyass A, Moustaghfir A, Zbir E, Hda A, Hamani A	2011
Acute necrotizing pancreatitis complicated with ST elevation acute myocardial infarction: a case report and literature review	Hsu PC, Lin TH, Su HM, Lin ZY, Lai WT, Sheu SH	2010
Perplexing epigastric pain-coincident myocardial infarction and acute pancreatitis	Wu CH, Wang KL, Lu TM	2010
T-wave depletion and bradycardia possibly secondary to acute pancreatitis: review of the literature	Türkay C, Aydoğan T, Karanfil A, Uyar ME, Selçoki Y, Kanbay M	2009
Deceived by acute pancreatitis masquerading as acute inferior myocardial infarction	Low TT, Lee LC, Lee CH	2009
Emergency treatment succeeded in acute myocardial infarction with acute pancreatitis and multi-organ failure: a case report	Chen X, Lu CZ, Li C	2008
Acute pancreatitis presenting as acute inferior wall ST-segment elevations on electrocardiography	Makaryus AN, Adedeji O, Ali SK	2008
Acute pancreatitis mimicking acute inferior myocardial infarction	Tejada JG, Hernández F, Chimeno J, Alonso MA, Martin R, Bastante T	2008
Acute pancreatitis mimicking acute myocardial infarction or vice versa? An EKG case report	Makowska E, Czempik E	2005
ST-segment elevation pattern and myocardial injury induced by acute pancreatitis	Korantzopoulos P, Pappa E, Dimitroula V, Kountouris E, Karanikis P, Patsouras D, Siogas K	2005

Diagnosis of acute myocardial infarction in a context of acute pancreatitis is a challenging task. ECG changes and elevated cardiac troponins due to rhabdomyolysis can mislead towards the diagnosis of acute myocardial infarction. Subsequent treatment particularly with thrombolytic therapy can have disastrous out come in a patient with acute pancreatitis. In such a context an urgent coronary angiogram would have a pivotal role to decide on exact diagnosis and management. Management of such a patient is also challenging with the choice of revascularization therapy and safety of antiplatelet agents and anticoagulant therapy. Therefore these issues need further evaluation based on research and evidence.

## Abbreviations

ECG: electrocardiogram
2D-echo: 2 dimensional echocardiogram
CT: computerized tomography
CPK: creatinine phosphokinase
